# Narrative Review of the Pathophysiology of Post-Infectious Bronchiolitis Obliterans

**DOI:** 10.3390/biom16071061

**Published:** 2026-07-20

**Authors:** Alessandro Zago, Caterina Cocchi, Massimo Maschio, Laura Badina, Francesca Policastro, Alessandro Amaddeo, Egidio Barbi, Sergio Ghirardo

**Affiliations:** 1 Department of Medicine, Surgery and Health Sciences, University of Trieste, 34137 Trieste, Italy; dr.alessandrozago@gmail.com (A.Z.); cate160cocchi@gmail.com (C.C.); egidio.barbi@burlo.trieste.it (E.B.); sergio.ghirardo@burlo.trieste.it (S.G.); 2 Department of Pediatric, Institute for Maternal and Child Health-IRCCS Burlo Garofolo, 34137 Trieste, Italy; massimo.maschio@burlo.trieste.it (M.M.); laura.badina@burlo.trieste.it (L.B.); francesca.policastro@burlo.trieste.it (F.P.)

**Keywords:** post-infectious bronchiolitis obliterans, bronchiolitis obliterans, narrative review, airway remodeling, pulmonary fibrosis

## Abstract

Post-Infectious Bronchiolitis Obliterans (PIBO) is a rare chronic obstructive lung disease characterized by irreversible airflow limitation due to inflammation, fibrosis, and obliteration of the small airways following severe lower respiratory tract infection. Adenovirus is the most frequently associated pathogen, followed by other viruses and atypical bacteria. PIBO pathogenesis appears to result from a complex interaction between severe epithelial injury, dysregulated immune response, persistent neutrophilic inflammation, abnormal tissue repair, and genetic susceptibility. Epithelial damage triggers the release of inflammatory cytokines and epithelial-derived alarmins, promoting chronic inflammation and airway remodeling through fibrosis and airway obliteration driven by activation of the TGF-β/CTGF pathway, epithelial–mesenchymal transition, macrophage–fibroblast interactions, and extracellular matrix deposition. Genetic factors affecting mucociliary clearance, innate immunity, and fibrotic pathways may further predispose certain individuals to abnormal repair and fibrosis consequently. Histologically, PIBO progresses from inflammatory bronchiolitis to fibroproliferative remodeling and constrictive bronchiolitis with luminal obliteration. Understanding these mechanisms supports a stage-based therapeutic approach targeting inflammation in early disease and fibrotic remodeling in advanced stages.

## 1. Introduction

Post-Infectious Bronchiolitis Obliterans (PIBO) is a rare chronic obstructive lung disease characterized by persistent inflammation and fibrosis of the small airways, leading to irreversible airflow limitation with a global negative impact on cardiorespiratory fitness [1]. The disease most commonly develops after severe lower respiratory tract infections during childhood, although similar pathological mechanisms may also occur after lung transplantation, hematopoietic stem cell transplantation, or toxic inhalation exposure. PIBO is characterized histologically by inflammation, submucosal fibrosis, and progressive narrowing or obliteration of the bronchiolar lumen.

Among infectious triggers, adenovirus, particularly genotypes 3, 7, and 21, is the pathogen most frequently associated with PIBO, although other viruses and atypical bacteria such as influenza virus, respiratory syncytial virus, human metapneumovirus, *Mycoplasma pneumoniae*, *Bordetella pertussis*, and SARS-CoV-2 have also been implicated. Despite increasing recognition of the disease, the exact mechanisms leading from acute infection to chronic airway obliteration remain incompletely understood.

The development of PIBO appears to result from a complex interaction between epithelial injury, dysregulated immune response, persistent inflammation, abnormal tissue repair, and genetic susceptibility, such as gene encoding proteins involved in ciliar motility and mucous clearance (DNAH9, DNAH1, and MUC5). Understanding these mechanisms is crucial for identifying risk factors, early diagnostic markers, and potential therapeutic targets. Therefore, this narrative review aims to summarize current knowledge on the pathogenetic mechanisms of PIBO, focusing on infectious triggers, immune dysregulation, epithelial injury, airway remodeling, and genetic predisposition.

## 2. Infectious Triggers

Severe lower respiratory tract infections represent the main initiating event in PIBO. The pathogens most associated with PIBO include respiratory viruses such as adenovirus, respiratory syncytial virus (RSV), influenza virus, parainfluenza virus, rhinovirus, and human metapneumovirus, as well as atypical bacteria such as *Mycoplasma pneumoniae*. Among these, adenovirus has historically been considered the pathogen most strongly associated with PIBO development, particularly in pediatric populations [2,3,4].

The severity of the initial infection appears to play a major role in the development of PIBO. Several studies have shown that children who develop PIBO are more likely to have experienced severe pneumonia characterized by hypoxemia, prolonged fever, need for mechanical ventilation, long hospital stay, and extensive pulmonary consolidation on high-resolution computed tomography (HRCT). Laboratory findings such as elevated lactate dehydrogenase (LDH), neutrophilia, and increased inflammatory markers have also been associated with an increased risk of PIBO. These findings suggest that the intensity of the inflammatory response and the extent of epithelial injury, rather than the specific pathogen alone, are key determinants in the progression toward chronic airway obstruction [5,6].

Coinfections may further increase the risk of PIBO development. In particular, viral coinfections or coinfection with *Mycoplasma pneumoniae* have been associated with more severe pneumonia and prolonged inflammatory response. Coinfections may amplify immune activation, increase epithelial damage, and delay tissue repair, thereby promoting airway remodeling and fibrosis. Some studies have suggested that coinfections are associated with higher inflammatory markers, longer duration of fever, and more extensive radiological involvement, all of which are recognized risk factors for PIBO [2,7].

Emerging epidemiological data support a post-pandemic rise in PIBO cases, although this increase appears to be driven primarily by the resurgence of known PIBO-associated pathogens rather than by SARS-CoV-2 itself. A single-center Spanish study documented a nearly tenfold increase in PIBO cases during 2022–2023 compared with pre-pandemic years, following a complete absence of cases during 2020–2021 [8].

This rebound is largely attributable to the so-called “immunity debt” phenomenon: the prolonged suppression of endemic respiratory viruses resulted in an expanded pool of immunologically naïve children who lacked both primary and boosted pathogen-specific immunity [9]. The resurgence of multiple respiratory viruses in older and more susceptible pediatric cohorts led to more severe lower respiratory tract infections and PIBO as a consequence [10].

Except for isolated case reports of PIBO following COVID-19 [11], the post-pandemic rise in PIBO should be explained by viral and immunological changes indirectly induced by the pandemic.

The recent literature also suggests that the epidemiology of PIBO infectious triggers may vary significantly across geographic regions. In particular, a growing body of evidence from China indicates an increasing proportion of PIBO cases developing after *Mycoplasma pneumoniae* pneumonia, whereas European cohorts still report adenovirus as the predominant etiological agent. Several large Chinese cohort studies have reported that severe *Mycoplasma pneumoniae* pneumonia can lead to PIBO, particularly in children presenting with extensive pulmonary consolidation, prolonged fever, elevated inflammatory markers, and persistent radiological abnormalities. In these patients, airway injury appears to be mediated not only by direct pathogen damage but also by an exaggerated immune response, with high levels of inflammatory cytokines and neutrophilic inflammation contributing to airway remodeling and fibrosis [12].

Conversely, European studies continue to identify adenovirus as the most common infectious trigger associated with PIBO. Adenovirus infections are known to cause severe necrotizing bronchiolitis, epithelial destruction, and prolonged viral persistence, all of which may contribute to abnormal airway repair and fibrotic remodeling. Compared with other pathogens, adenovirus infections are more frequently associated with mechanical ventilation, prolonged hospitalization, and multifocal pneumonia, all of which are recognized risk factors for PIBO development. Moreover, adenovirus-related PIBO has been associated with higher levels of inflammatory cytokines and worse respiratory outcomes compared with PIBO caused by other pathogens [13,14,15].

The reasons for these geographic differences remain unclear.

Overall, infectious triggers initiate the cascade of events that lead to epithelial injury, immune dysregulation, persistent inflammation, and airway fibrosis, which represent the key pathogenetic mechanisms underlying PIBO.

## 3. Pathogenic Mechanisms

### 3.1. Epithelial Injury and Impaired Repair

The initial step in PIBO pathogenesis is believed to involve severe injury to the bronchiolar epithelium caused by infection and inflammation. Viral infections, particularly adenovirus, can cause epithelial necrosis, destruction of the mucosal barrier, and the formation of intranuclear inclusion bodies. This extensive damage leads to exposure of the basement membrane and the potential activation of inflammatory and fibrotic pathways [3,16,17].

Normally, epithelial injury is followed by regeneration and restoration of normal airway structure. However, in PIBO, epithelial repair appears to be abnormal or incomplete, although the exact downstream checkpoints remain poorly defined in pediatric tissue. Persistent epithelial damage is hypothesized to trigger epithelial–mesenchymal transition (EMT), a process in which epithelial cells acquire mesenchymal characteristics and contribute to fibroblast proliferation and extracellular matrix deposition. This hypothesis applies the mechanism encompassed by the umbrella term epithelial–mesenchymal transition (EMT), which can be classified into three types according to its involvement in embryogenesis, wound healing and tissue fibrosis, and neoplastic cell proliferation.

By analogy with more extensively studied chronic airway diseases, as described in murine models of asthma and chronic obstructive pulmonary disease, it has been shown that loss of E-cadherin expression, increased expression of mesenchymal markers such as vimentin and S100A4, and increased matrix metalloproteinase activity are associated with airway remodeling and fibrosis. While similar mechanisms are frequently presumed to play a parallel role in PIBO, direct histological and molecular confirmation in children remains limited, leaving the exact trajectory from epithelial shedding to fixed luminal narrowing partly speculative [18].

In recent years, increasing attention has been given to the potential role of epithelial-derived cytokines, particularly thymic stromal lymphopoietin (TSLP), in the link between epithelial injury and chronic airway inflammation and remodeling. In particular, it was described in the dysfunction of barrier integrity not only in asthma and other allergic conditions, but also in atopic dermatitis, intestinal bowel diseases, and malignancy. TSLP is an epithelial-derived alarmin upregulated during viral infections, epithelial damage, and environmental stimuli, and it plays a central role in initiating type 2 and non-type 2 inflammatory responses. Following viral infection and epithelial injury, damaged epithelial cells release TSLP together with other alarmins such as IL-25 and IL-33, which activate dendritic cells, innate lymphoid cells, and T lymphocytes, promoting a persistent inflammatory response. TSLP has been shown in the literature to promote Th2 polarization, increase production of IL-4, IL-5, and IL-13, and enhance eosinophilic and neutrophilic inflammation. In addition to these immunomodulatory effects described in other chronic respiratory disorders, it is hypothesized that TSLP might contribute to airway remodeling by promoting fibroblast activation, collagen deposition, and smooth muscle proliferation. Increased TSLP expression has been described in several chronic airway diseases characterized by airway remodeling and fibrosis, suggesting that epithelial-derived cytokines may represent a key link between epithelial injury and fibrotic airway remodeling. To the best of our knowledge, no specific studies on TSLP pathways in PIBO are currently available. Nevertheless, although highly speculative, the pathogenic mechanisms involved in PIBO, viral epithelial injury, persistent inflammation, and airway fibrosis, closely overlap with known TSLP-driven processes, suggesting that TSLP may play a role in the transition from acute epithelial damage to chronic airway remodeling and obliteration [19,20,21].

### 3.2. Inflammation and Immune Dysregulation

Inflammation plays a central role in the development of PIBO. Several studies have shown that patients who develop PIBO after severe pneumonia exhibit a specific inflammatory profile characterized by altered levels of cytokines such as IL-2, IL-6, IL-10, TNF-α, IFN-γ, and IL-18. These cytokines are involved in T-cell activation, macrophage activation, regulation of antiviral immune responses, and tissue repair processes. The balance between pro-inflammatory and regulatory cytokines appears to be a key determinant in whether airway inflammation resolves or progresses toward chronic remodeling and fibrosis [16,22,23].

Recent evidence suggests that cytokine dysregulation rather than simple hyperinflammation may play a major role in PIBO pathogenesis. Reduced levels of IL-18 observed in some PIBO cohorts may be particularly relevant, as IL-18 promotes Th1 polarization and IFN-γ production, which are important for viral clearance and resolution of inflammation. Decreased IL-18 activity may theoretically impair effective antiviral immune responses and shift the immune balance toward Th17-mediated immunity, which is strongly associated with neutrophilic inflammation through IL-17-mediated induction of IL-8 and neutrophil recruitment. This proposed mechanism could contribute to the persistent neutrophilic inflammation frequently observed in PIBO. Interestingly, conditions characterized by high IL-18 levels, such as systemic juvenile idiopathic arthritis-associated lung disease, are more commonly associated with interstitial and alveolar fibrosis rather than bronchiolar fibrosis, supporting the hypothesis that different cytokine profiles may lead to distinct patterns of lung injury and fibrosis [24,25].

IL-2 also appears to play a role in PIBO pathogenesis through its effects on T-cell proliferation and immune regulation. IL-2 is a key cytokine for T-cell expansion and survival, particularly regulatory T-cells (Tregs), which are essential for controlling excessive immune responses and limiting tissue damage. Dysregulation of IL-2 signaling could potentially lead to impaired regulatory T-cell function and prolonged inflammation, thereby favoring chronic airway injury and fibrosis. Therefore, IL-2 may play a dual role in PIBO pathogenesis by influencing the balance between immune activation and immune regulation [3,23,26].

IL-6 is another cytokine frequently elevated during severe respiratory infections and has been associated with worse outcomes and prolonged inflammation. IL-6 is a pleiotropic cytokine involved in acute-phase response, B-cell activation, T-cell differentiation, and fibroblast activation. In general models of fibrotic lung diseases and airway remodeling, chronic elevation of IL-6 has been associated with progressive damage, as IL-6 can promote fibroblast proliferation, collagen synthesis, and smooth muscle cell proliferation. IL-6 may therefore represent a key mediator linking acute inflammation to chronic airway remodeling and fibrosis in PIBO, although direct tissue confirmation in pediatric cohorts remains limited. Additionally, IL-6 may contribute to the persistence of inflammation by promoting Th17 differentiation and neutrophilic inflammation, both of which are associated with chronic airway injury [12,27,28].

IL-10, traditionally considered an anti-inflammatory cytokine, may also have a complex role in PIBO pathogenesis, despite the fact that it has not been described in human models. The literature describes evidence about its role in animal models of intestinal bowel disease, some forms of hepatitis (Concavaline and Galactosamine-induced) and experimental autoimmune encephalomyelitis. While IL-10 reduces acute inflammatory responses by inhibiting macrophage and T-cell activation, chronic elevation of IL-10 has been associated with fibrotic processes in several organs, including the lungs. Data extrapolated form related fibrotic models suggest that IL-10 may promote fibrosis indirectly by suppressing effective pathogen clearance and prolonging low-grade inflammation, and directly by promoting fibroblast survival and extracellular matrix deposition in certain contexts. Some studies have suggested that IL-10 may contribute to fibrotic remodeling by shifting the immune response toward a regulatory and profibrotic environment rather than a purely inflammatory one. Therefore, IL-10 represents another potential contributor to the transition from acute inflammation to chronic fibrosis in PIBO [16,29].

Recent multicenter data gathered from 24 children with PIBO have further expanded the serum and BALF cytokine profiling in pediatric PIBO. Specifically, IL-27 has been identified as a dysregulated biomarker in the BALF of children with PIBO, correlating with the severity of post-viral small-airway obstruction [30]. Furthermore, epigenetic regulators have emerged as promising circulatory markers; recent data demonstrated that micro-RNA-766-3p is altered in the serum of PIBO patients. Additionally, serum levels of YKL-40, a well-known marker of macrophage activation and structural remodeling, was found to reflect ongoing subepithelial fibrotic activity and tracked closely with long-term lung function in affected children, as explored in a retrospective study involving 37 children with PIBO exacerbation [31].

Neutrophilic inflammation appears to be particularly important in PIBO pathogenesis. Elevated neutrophil counts and increased levels of inflammatory markers such as lactate dehydrogenase have been associated with increased risk of PIBO. Extrapolating from the general airway-remodeling literature, neutrophils release proteases, reactive oxygen species, and matrix metalloproteinases, which can contribute to tissue damage, extracellular matrix degradation, and airway remodeling [32].

Other inflammatory mediators such as IL-8, myeloperoxidase (MPO), and matrix metalloproteinase (MMP), in particular MMP-9, have also been implicated in airway inflammation and remodeling, as they are directly detected in BAL [33,34].

Experimental models have shown that inhibition of these mediators can reduce airway hyperresponsiveness, inflammatory cell infiltration, and fibrosis, supporting their potential pathogenic role in chronic airway obstruction [33,35,36]. In particular, high levels of MMP-9 were found in the broncho-alveolar fluid; however its role in pathogenesis is controversial, as a recent study on an ex vivo epithelial model showed no difference in the molecule levels in PIBO models versus controls.

Innate immunity may also play a role in PIBO development. Mannose-binding lectin (MBL), a component of the complement lectin pathway, has been associated with increased susceptibility to PIBO. MBL deficiency or polymorphisms, as detected in a cohort of patients affected by PIBO, may impair pathogen clearance and prolong inflammation, thereby increasing the theoretical risk of chronic airway damage and airway remodeling [37].

Translational studies have highlighted a dysregulation of epithelial-derived antimicrobial peptides in the BALF of children with PIBO. Specifically, a significant downregulation of Cathelicidin (LL-37) and *beta2*-defensin was observed in the BALF during chronic stages. This local deficiency may impair mucosal defense, favor subclinical viral persistence and fuel the chronic, neutrophil inflammatory loop that drives progressive bronchiolar obliteration [38].

Overall, immune dysregulation in PIBO appears to involve a complex and dynamic imbalance between pro-inflammatory, regulatory, and profibrotic cytokines, which may determine whether acute airway injury successfully resolves or progresses toward chronic bronchiolar fibrosis and airway obliteration.

### 3.3. Fibrosis and Airway Obliteration

PIBO fibrosis is the result of a potentially self-reinforcing TGF-β/CTGF-driven aberrant repair program. Direct pediatric PIBO biomarker data are still limited, but the available evidence points in that direction, and the broader BO/BOS literature gives the pathway biologic credibility. In children with PIBO, a key proteomic study including 15 PIBO cases, 18 asthma cases, and 30 healthy controls identified TGF-β1 as a PIBO-associated biomarker; furthermore, higher systemic TGF-β1 was negatively correlated with lung-function indices including forced expiratory volume in the first second (FEV1), FEV1/forced vital capacity (FVC) ratio, and maximal mid-expiratory flow, linking the profibrotic signal to fixed airflow obstruction [39]. In parallel, BO/BOS studies have shown that the fibrotic airway milieu is reflected locally in bronchoalveolar lavage: in one classic BAL study, epithelial lining fluid from BOS patients showed markedly higher IL-8 (52.4 ± 22.2 vs. 4.4 ± 0.9 pg/mL) and TGF-β (5.6 ± 1.9 vs. 0.9 ± 0.2 ng/mL) than controls, supporting the hypothesis that neutrophilic inflammation and TGF-β-driven fibroproliferation might coexist in the diseased small airway of PIBO patients [40].

Mechanistically, TGF-β is well positioned to couple epithelial injury to irreversible remodeling: based on established models of lung fibrosis, after epithelial denudation, activated macrophages, epithelial cells, and probably fibroblasts themselves sustain local TGF-β signaling. This axis promotes fibroblast recruitment, differentiation into α-SMA-positive myofibroblasts, extracellular matrix production and epithelial–mesenchymal transition. In BO models and human BO/BOS tissue, airway fibrosis has also been linked to macrophage-derived growth factor programs, including platelet-derived growth factor (PDGF) and high-airway TGF-β activity, which can amplify recruitment of profibrotic monocyte/macrophage populations and perpetuate tissue remodeling [41].

A particularly relevant downstream mediator in fibrotic diseases is CTGF/CCN2, whose function was analyzed in post-transplantation lung fibrosis, which functions as a canonical TGF-β effector and stabilizer of fibrosis rather than a mere bystander. Although CTGF has not yet been systematically characterized in pediatric PIBO BAL, transplant-related pulmonary fibrosis studies show increased CTGF mRNA in diseased lungs, strong epithelial CTGF staining, and higher BAL CTGF protein in fibrotic phenotypes, supporting the hypothesis that once the TGF-β→CTGF axis is engaged, the repair response becomes self-maintaining [42]. Thus, in PIBO, the most plausible translated model is that persistent epithelial injury and neutrophil-rich inflammation create a local environment in which TGF-β initiates fibroblast activation, while CTGF consolidates this signal into durable matrix deposition, bronchiolar wall thickening, and patchy luminal obliteration. The key limitation remains that direct BAL-based TGF-β/CTGF evidence in pediatric PIBO is still scarce, meaning this specific cascade is largely inferred from serum/proteomic PIBO data and from mechanistically related BO/BOS settings rather than proven specifically in large clinical PIBO cohorts [39].

### 3.4. Persistent Inflammation and Chronic Remodeling

Even after resolution of the acute infection, persistent low-grade airway inflammation appears to contribute to ongoing airway remodeling and fibrosis in PIBO. Children who develop PIBO often show persistent respiratory symptoms and characteristic radiological abnormalities for months or years after the initial infection, supporting the concept that PIBO reflects a chronic dysregulated repair process rather than only residual acute injury. Bronchoalveolar lavage studies have shown persistent neutrophilic inflammation and increased IL-8 in PIBO, while more recent data also suggest upregulation of MMP-9 and MPO; however, these latter biomarker findings still require further validation in larger clinical trials [35].

One of the key mechanisms underlying chronic remodeling may be related to persistent epithelial injury and ongoing exposure to damage-associated antigens, possibly associated with delayed viral clearance or severe epithelial destruction. Persistent epithelial injury is hypothesized to be associated with the release of epithelial-derived alarmins such as thymic stromal lymphopoietin (TSLP), IL-33, and IL-25, which maintain activation of dendritic cells, innate lymphoid cells, and T lymphocytes as demonstrated for other conditions. This potentially results in a chronic inflammatory microenvironment characterized by neutrophilic inflammation, macrophage activation, and fibroblast stimulation. The inflammatory process is likely to become a self-perpetuating response that progressively leads to fibrosis and airway obliteration [2,16,43,44,45,46,47].

Macrophages are also likely to play an important role in chronic airway remodeling in PIBO, based on data extrapolated from related fibrotic networks. In chronic airway injury, macrophages can shift toward a profibrotic phenotype and produce mediators such as TGF-β, PDGF, CTGF, and other cytokines that promote fibroblast proliferation and extracellular matrix deposition. This macrophage–fibroblast interaction may represent one of the key mechanisms sustaining fibrosis even in the absence of active infection. In bronchiolitis obliterans syndrome (BOS) and other fibrotic airway diseases, macrophage-derived growth factors and TGF-β signaling have been associated with airway fibrosis and disease progression, suggesting that parallel cellular crosstalk might contribute to airway remodeling in PIBO [45,48,49,50,51,52,53].

Epithelial–mesenchymal transition (EMT) represents another putative mechanism that is hypothesized to contribute to chronic airway remodeling. EMT has been demonstrated in idiopathic pulmonary fibrosis, where TGF-β induces alveolar epithelial cells to acquire mesenchymal characteristics and contribute to myofibroblast populations. EMT has also been implicated in BOS after lung transplantation, where airway epithelial cells undergo phenotypic transition associated with airway fibrosis. These findings suggest that similar mechanisms could theoretically contribute to airway remodeling and fibrosis in PIBO. EMT may contribute to airway wall thickening, smooth muscle hypertrophy, and extracellular matrix deposition observed in PIBO [54,55,56].

The persistence of inflammation and airway remodeling may also be supported by ongoing immune dysregulation following the initial infection. Altered cytokine profiles, including increased pro-inflammatory and profibrotic cytokines such as IL-6 and persistent expression of immunoregulatory cytokines such as IL-10, may impair complete resolution of inflammation and promote the persistence of an inflammatory–profibrotic environment. In this context, the local immune response may shift from pathogen clearance toward tissue remodeling and fibrosis, potentially contributing to progressive airway obstruction [36,41].

**Figure 1:**
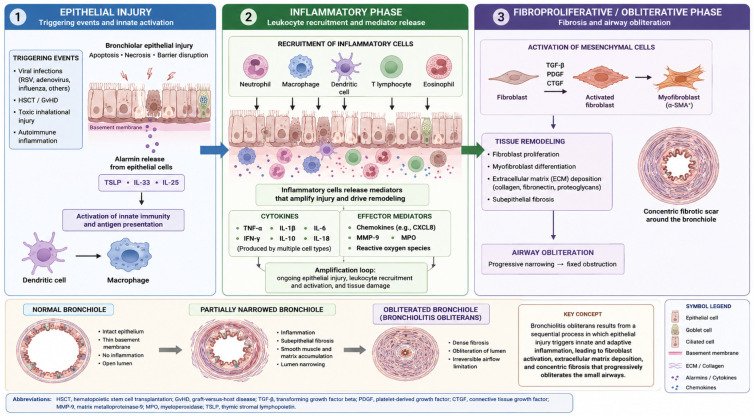
Pathogenetic mechanisms of Post-Infectious Bronchiolitis Obliterans. Illustrative figure generated using BioRender. Zago, A. (2026) https://BioRender.com/avhklrx (accessed on 11 July 2026).

Overall, PIBO may therefore be considered a disease of abnormal tissue repair rather than persistent infection alone. The postulated sequence of events likely involves severe epithelial injury, persistent neutrophilic inflammation, chronic activation of macrophages and fibroblasts, activation of TGF-β and CTGF pathways, potential epithelial–mesenchymal transition, and progressive extracellular matrix deposition, ultimately resulting in bronchiolar fibrosis and airway obliteration (Table 1, Figure 1). This chronic inflammatory–fibrotic loop represents a highly plausible explanation for why a subset of patients continues to show progression of airflow limitation even years after their initial infection has clinically resolved.

## 4. Genetic Susceptibility

Genetic predisposition may play an important role in determining which patients develop PIBO after severe respiratory infection, as only a small proportion of children with severe pneumonia progress to PIBO. Several genetic variants associated with PIBO or bronchiolitis obliterans (BO) involve genes related to mucociliary clearance, epithelial barrier function, innate immunity, cytokine regulation, and fibrotic pathways.

Mutations in genes involved in mucociliary clearance, such as DNAH9, DNAH1, and other primary ciliary dyskinesia-related genes, may impair pathogen and debris clearance, leading to prolonged infection, persistent inflammation, and increased risk of chronic airway injury and remodeling. Variants in mucin genes such as MUC5B, that have been found in small cohort of patients with the characteristic ground glass radiological phenotype, may alter epithelial stability, mucus properties, and repair processes, predisposing them to persistent airway injury and abnormal remodeling [57,58]. However, further physiological studies are required to confirm the role of those genes.

Polymorphisms have been observed in innate immunity genes, including mannose-binding lectin (MBL), as described in the study by Giubergia et al. involving one hundred and eleven patients [37], and NOD2/CARD15, which was evaluated in a case–control study of a large population of 181 donor–recipients pairs, in which recipients with the mutation developed BO with higher frequency, which may impair pathogen recognition and clearance and modify inflammatory responses.

Overall, PIBO likely results from the interaction between severe infectious epithelial injury and a genetically determined abnormal repair response characterized by persistent inflammation and exaggerated fibrotic remodeling, leading to irreversible bronchiolar obstruction.

## 5. Histopathology of Post-Infectious Bronchiolitis Obliterans

**Figure 2:**
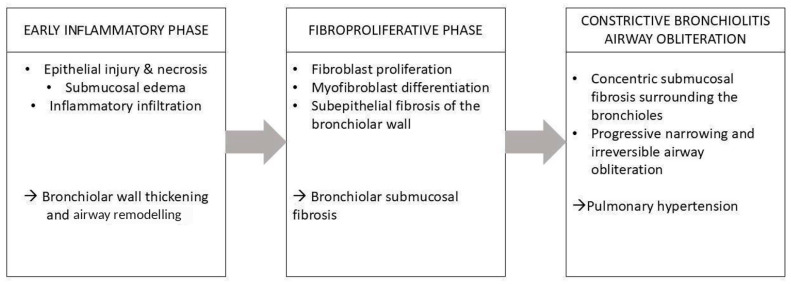
Stepwise pathophysiological trajectory of Post-Infectious Bronchiolitis Obliterans.

PIBO is histologically characterized by inflammation, fibrosis, and progressive obliteration of the small airways. The pathological process primarily involves membranous and respiratory bronchioles and may include different stages of airway injury and repair, ranging from active inflammatory bronchiolitis to fibroproliferative lesions and established fibrotic obliteration (Figure 2). Histological changes are typically patchy and heterogeneously distributed, which explains why lung biopsy may sometimes underestimate disease severity.

Two main histopathological patterns of BO have been described, the proliferative (inflammatory) form and the constrictive (fibrotic) form, although these likely represent different stages of the same pathological process rather than completely distinct entities [59,60,61].

Early inflammatory stage.

In the early stages of the disease, histology shows epithelial injury, epithelial necrosis, and inflammatory infiltration of the bronchiolar wall. The inflammatory infiltrate is typically composed of neutrophils, lymphocytes, and macrophages. The bronchiolar epithelium may appear denuded or ulcerated, and inflammatory exudates may be present within the bronchiolar lumen.

Submucosal edema and inflammatory cell infiltration lead to thickening of the bronchiolar wall and narrowing of the lumen. In this phase, the airway obstruction is mainly due to inflammation and edema rather than fibrosis, which explains why early anti-inflammatory therapy may still modify disease progression [2,62,63].

Fibroproliferative stage. 

As PIBO progresses, fibroproliferative remodeling becomes the predominant histopathological feature, characterized by fibroblast proliferation, myofibroblast differentiation, and progressive subepithelial fibrosis of the bronchiolar wall. Myofibroblasts produce extracellular matrix components, including collagen types I and III, fibronectin, and proteoglycans, leading to submucosal fibrosis, airway wall thickening, and progressive luminal narrowing. Transforming growth factor-β (TGF-β) is considered a key profibrotic mediator in most fibrosing lung diseases, promoting fibroblast proliferation, myofibroblast differentiation, and extracellular matrix deposition. CTGF, a downstream mediator of TGF-β signaling, may further amplify fibroblast activation and collagen deposition, contributing to progressive airway fibrosis and bronchiolar obliteration. Smooth muscle hypertrophy and hyperplasia may also occur in the bronchiolar wall, further contributing to fixed airway narrowing [44,63,64].

Constrictive bronchiolitis and airway obliteration.

In the late stages of PIBO, histology shows concentric submucosal fibrosis surrounding the bronchioles, leading to progressive narrowing and eventual obliteration of the airway lumen. This pattern is often referred to as constrictive bronchiolitis, which represents the most typical histological pattern in PIBO.

Luminal obliteration is usually patchy, and completely obliterated bronchioles may be found adjacent to relatively preserved airways. This patchy distribution explains the characteristic mosaic perfusion pattern seen on high-resolution computed tomography, which reflects areas of air trapping and regional hypoperfusion [59].

At this stage, as for other BO types, inflammation may be minimal, and the disease is dominated by fibrosis and airway remodeling. The airway lumen may be completely replaced by fibrous tissue, resulting in irreversible airflow obstruction [60].

Comparison with BOS.

Histologically, PIBO shares many features with BOS after lung or hematopoietic stem cell transplantation, including bronchiolar inflammation, submucosal fibrosis, smooth muscle hypertrophy, and luminal obliteration. These similarities suggest that different etiological triggers may lead to a common final pathway of airway fibrosis and obliteration mediated by epithelial injury, immune dysregulation, and fibrotic remodeling pathways [65].

## 6. Potential Therapeutic Targets

### 6.1. Established Clinical Practice

Anti-inflammatory and immunomodulatory therapies remain the cornerstone of current clinical management for PIBO, primarily because the disease evolves from an acute phase of intense epithelial injury and cytokine-driven airway inflammation. The clinical rationale is therefore to intervene before remodeling becomes fully irreversible. Within this framework, corticosteroids are the most widely used agents in daily practice, particularly during the early stages of the disease. However, their long-term benefit is uncertain, and prolonged systemic exposure is problematic in children because of growth, metabolic, skeletal, and infectious adverse effects. Reviews consistently describe steroid treatment as empirical rather than evidence-based, with the strongest rationale in the early inflammatory phase rather than in established scarred airways [65,66].

Within this anti-inflammatory framework, pulse intravenous methylprednisolone has attracted interest in obtaining high anti-inflammatory potency while reducing chronic daily steroid exposure. Older pediatric follow-up data suggested improvements in oxygenation, exacerbation burden, and hospitalization frequency in some children with PIBO treated with pulse methylprednisolone, and more recent observational work from France also reported a reduction in exacerbations and inhaled corticosteroid requirements after pulse treatment. At the same time, a 2025 retrospective monocentric cohort found no clear effect of methylprednisolone pulses on subsequent evolution compared with untreated severe cases, which highlights how dependent this literature still is on cohort selection, timing of intervention, and baseline disease severity [67,68].

Another therapeutic approach currently used in clinical practice is represented by inhaled corticosteroids, that may be more rational in patients who remain symptomatic but are no longer in the most florid inflammatory phase, especially because PIBO is a chronic small-airway disease in which ongoing local inflammation may still be pharmacologically modifiable. A 2022 study reported that inhaled corticosteroids improved lung function and relieved airway obstruction in patients older than 5 years in remission, with greater apparent benefit in those with positive bronchodilator testing, suggesting that some children retain a modifiable inflammatory/bronchomotor component even within a fixed obstructive phenotype. This supports a phenotype-based approach: children with recurrent wheeze, exacerbations, or partial reversibility may derive more benefit from inhaled therapy than those with advanced fixed fibro-obliterative disease [62].

### 6.2. Observational Evidence

Because PIBO increasingly looks like a disorder of dysregulated repair rather than a purely post-infectious scar by analogy with other chronic airway diseases, growing attention has been directed toward epithelial alarmins, altered T-cell signaling, macrophage activation, and a persistent neutrophil-rich inflammatory milieu which maintain remodeling after the infection has cleared. Therefore, treatment should not aim only to suppress inflammation globally but to reprogram it. This is the conceptual basis for using combination regimens that try to reduce inflammatory signaling while sparing systemic steroids. In this context, it should be pointed out that there should be some therapeutic combinations. The best studied pediatric example is the budesonide–azithromycin–montelukast–acetylcysteine approach, sometimes referred to as BAMA. In a 2021 follow-up study, this regimen was associated with improvement in symptoms, pulmonary function, and HRCT findings while also reducing systemic steroid use; the report described the strategy as clinically effective, but it remained observational and not randomized [69].

Macrolides, especially azithromycin, are among the most biologically appealing treatments because they target a central PIBO mechanism: persistent neutrophilic inflammation. Their benefit is probably not primarily antimicrobial in established PIBO but anti-inflammatory, through effects on neutrophil trafficking, IL-8 signaling, mucus hypersecretion, and possibly biofilm-related airway injury. In BOS after lung transplantation, azithromycin reduced airway neutrophilia and IL-8, giving mechanistic support to its use in neutrophil-predominant small-airway disease. In PIBO cohorts, low-dose azithromycin is repeatedly associated with symptom improvement and is commonly used in combination with steroids or inhaled therapies, although robust randomized pediatric PIBO data are lacking [65,70,71].

The pediatric PIBO data for azithromycin are modest but directionally consistent. A 2015 clinical series reported that children treated with low-dose azithromycin plus prednisone improved clinically in 10 of 16 followed cases, although HRCT changes were limited and not all patients responded. A 2020 study in children under 5 years found that combination therapy with budesonide, montelukast, and azithromycin given for at least 3 months improved tidal pulmonary function indices and symptom scores compared with more irregular treatment, and the 2021 BAMA follow-up suggested that these benefits might be sustained while reducing systemic steroid exposure. Collectively, these data support azithromycin as a reasonable background therapy in PIBO, particularly in patients with exacerbations, chronic wet cough, or evidence of neutrophil-dominant airway inflammation [69,70,71].

### 6.3. Experimental and Hypothetical Future Approaches

Cytokine-targeted therapies are conceptually one of the most important future directions because the molecular model of PIBO increasingly implicates specific immune pathways rather than nonspecific inflammation. None of the following approaches have been tested in patients with PIBO. These proposals are based solely on disease analogy and speculation regarding shared biological mechanisms. In this framework, candidate nodes would include epithelial alarmins such as TSLP which has been demonstrated to be key in viral infections and chronic fibrotic conditions, downstream cytokines such as IL-6, and profibrotic mediators such as TGF-β. At present, though, direct interventional PIBO data are essentially absent. The case for these therapies is therefore mechanistic: if PIBO is driven by epithelial damage, alarmin release, persistent macrophage and T-cell activation, and fibroblast recruitment, then biologics targeting those axes could be most effective early, before fixed airway obliteration is established. This is especially compelling for children with biomarker evidence of ongoing inflammatory activity despite standard therapy [36,65].

Among cytokine and fibrotic signaling pathways, several molecular targets have emerged as potentially relevant in PIBO. IL-6 represents a credible target because it links acute inflammation to chronic remodeling by promoting Th17 differentiation, neutrophil persistence, fibroblast activation, and extracellular matrix deposition. Thymic stromal lymphopoietin (TSLP), an epithelial alarmin released after viral epithelial injury, may also contribute to early immune activation and airway remodeling. However, TGF-β appears to play the central role in airway fibrosis by promoting fibroblast proliferation, myofibroblast differentiation, epithelial–mesenchymal transition, and collagen deposition. Because direct inhibition of TGF-β is difficult due to its broad physiological functions, downstream mediators such as connective tissue growth factor (CTGF) may represent more feasible therapeutic targets. CTGF promotes fibroblast proliferation, extracellular matrix deposition, and tissue remodeling, and inhibition of CTGF has shown antifibrotic effects in pulmonary fibrosis, suggesting that targeting the TGF-β/CTGF axis may be a rational therapeutic strategy in fibro-obliterative airway diseases.

Anti-fibrotic therapy represents an important unmet need in PIBO because irreversible airway obstruction reflects established fibroproliferative remodeling in addition to persistent inflammation. Profibrotic cytokines such as TGF-β have been associated with lung function impairment, and bronchoalveolar lavage studies in BOS have demonstrated increased TGF-β together with IL-8, suggesting that neutrophilic inflammation and fibroblast-activating signals coexist in diseased small airways. This supports the hypothesis that airway fibrosis in PIBO results from the interaction between persistent neutrophilic inflammation and activation of profibrotic pathways such as TGF-β and CTGF.

Antifibrotic agents developed for interstitial lung diseases have recently been investigated in pediatric fibrosing lung diseases and BOS. Nintedanib has been evaluated in children with fibrosing interstitial lung disease in the InPedILD trial, showing acceptable safety and suggesting a reduction in lung function decline. Pirfenidone has also been studied in BOS after hematopoietic stem cell transplantation, where it was safe and associated with stabilization of lung function in some patients. Although PIBO is primarily a small-airway disease, the underlying fibrotic pathways share similarities with other fibrotic lung diseases, suggesting that antifibrotic therapies such as nintedanib or pirfenidone may represent potential therapeutic options in selected PIBO patients with progressive airway remodeling. However, these drugs have not yet been specifically validated in pediatric PIBO, and their potential role is largely extrapolated from interstitial lung disease and BOS.

Overall, these findings suggest that in patients with chronically progressive forms of PIBO future therapeutic strategies may need to target not only inflammation but also fibrotic remodeling pathways to slow down progression toward irreversible airway obstruction [72,73,74,75,76].

Matrix metalloproteinase inhibition is another biologically coherent strategy because MMP-driven matrix turnover appears to perpetuate epithelial damage and aberrant repair in PIBO. Experimental and translational studies have implicated MMP-9, IL-8, and myeloperoxidase in airway remodeling, and inhibition of these pathways in models can reduce inflammatory infiltration, airway hyperresponsiveness, and fibrosis. The challenge is that MMPs are involved in both injury and normal repair, so broad inhibition could be double-edged. Still, in a disease marked by neutrophil-rich protease activity, selective modulation of protease networks remains an attractive target class, particularly if paired with biomarker-guided patient selection [77,78].

Therapies targeting epithelial repair and regeneration may ultimately be the most disease-modifying approach because PIBO begins with severe epithelial injury and appears to persist when repair becomes aberrant. In this model, the aim is not only to suppress inflammation or fibrosis, but to restore epithelial barrier integrity, normalize epithelial–mesenchymal signaling, and reestablish functional mucociliary clearance. This could include strategies that reduce ongoing epithelial stress, block EMT-promoting signals, or improve airway epithelial regeneration. These approaches are not clinically established in PIBO yet, but they fit the pathogenesis better than late anti-inflammatory escalation in already scarred bronchioles [79,80]. A comprehensive overview of therapeutic targets is provided in Table 2.

The current therapeutic landscape suggests a stage-based and mechanism-based model. In early or active disease, anti-inflammatory and immunomodulatory therapy is most rational, particularly methylprednisolone boluses [67,81], inhaled corticosteroids, montelukast and azithromycin-based combinations. In patients with recurrent exacerbations and evidence of neutrophilic activity, macrolides appear especially attractive. In patients with progressive fixed obstruction despite control of acute symptoms, the therapeutic question shifts toward anti-fibrotic and epithelial-repair strategies, although these remain investigational. This staging logic may be more useful than asking whether a single drug “works” for all PIBO, because the disease likely evolves from inflammation-dominant to fibrosis-dominant phases.

## 7. Conclusions

PIBO is a chronic and largely irreversible lung disease resulting from severe bronchiolar epithelial injury followed by persistent inflammation and dysregulated repair processes leading to fibroproliferative remodeling and airway obliteration. Its pathogenesis involves a complex interaction between infectious injury, immune dysregulation, epithelial damage, profibrotic signaling pathways, and genetic susceptibility. We propose a conceptual framework in which PIBO may be divided into an early predominantly inflammatory phase and a later predominantly fibrotic phase, with disease behavior that may be either progressive or stable. Such a pathophysiological classification may help guide therapeutic strategies targeting inflammation in early disease and fibrotic remodeling in later stages.

## Figures and Tables

**Table 1:** Pathogenetic mechanisms and molecules.

Process	Molecules	Mechanism and Consequences
Epithelial Injury and Impaired Repair	↓ E-cadherin expression ↑ vimentin expression ↑ S100A4 ↑ matrix metalloproteinase activity Thymic stromal lymphopoietin (TSLP)	Damage of the basement membrane and exposure of the extracellular matrix Epithelial–mesenchymal transition (EMT)
Inflammation and Immune Dysregulation	↑ IL-2, ↑IL-6, ↑ IL-10, ↑ TNF-α, ↑ IFN-γ, and ↓ IL-18	T-cell activation, macrophage activation, upregulation of inadequate antiviral immune responses
	Reduced levels of IL-18 ↑ IL-17 ↑ IL-8	Persistent neutrophilic inflammation of the extracellular matrix leading to subepithelial fibrosis
	Innate immunity Mannose-binding lectin (MBL) deficiency	Impaired lectin pathway activation and opsonophagocytosis
	Dysregulation of IL-2 signaling	Impaired regulatory T-cell function and prolonged inflammation
	IL-6	Acute-phase response, B-cell activation, T-cell differentiation
	IL-10	Reduction in inflammation, viral persistence, fibroblast activation and collagen synthesis
	IL-8, myeloperoxidase (MPO)	Persistence of inflammation by promoting Th17 differentiation and neutrophilic inflammation
	Matrix metalloproteinase-9 (MMP-9)	May have a role in promoting fibroblast survival
Fibrosis and Airway Obliteration	TGF-β signaling	Fibroblast recruitment and extracellular matrix production
	CTGF/CCN2	TGF-β effector and stabilizer of fibrosis

↑ indicates upregulation/increased expression; ↓ indicates downregulation/decreased expression.

**Table 2:** Therapeutic targets.

Category	Target	Molecules	Mechanism
Anti-inflammatory	Acute inflammatory phase	Methylprednisolone pulses	Suppression of cytokine production, leukocyte recruitment, and epithelial inflammatory signaling
	Chronic inflammatory phase	Inhaled corticosteroids	Control of ongoing eosinophilic inflammation of small airways
	Persistent neutrophilic airway inflammation	Macrolides (azithromycin)	Inhibition of neutrophil trafficking, IL-8 signaling, mucus hypersecretion, biofilm-related airway injury
	Chronic inflammatory phase	BAMA: budesonide–azithromycin–montelukast–acetylcysteine approach	May help fluidification/mucoregulatory activity of bronchial secretions; limited evidence
Anti-fibrotic	TGF-β CTGF	Nintedanib Pirfenidone Pamrevlumab	An experimental drug used in clinical practice: Inhibition of fibroblast-activating signals that coexist with neutrophilic inflammation A future approach: IgG4 binding CTGF that may inhibit the final effector of the fibrosis
Matrix metalloproteinase inhibition	Inflammatory infiltration, airway hyperresponsive, fibrosis	Doxycicline	A hypothetical future approach to prevent MMP-driven matrix turnover and aberrant repair

## Data Availability

All data are available upon reasonable request to the authors.

## Abbreviations

BAMA (Budesonide–Azithromycin–Montelukast–Acetylcysteine); BO (Bronchiolitis Obliterans); BOS (Bronchiolitis Obliterans Syndrome); CARD15 (Caspase Recruitment Domain-containing protein 15); CCN2 (Cellular Communication Network factor 2 (also known as CTGF); COVID-19 (Coronavirus Disease 2019); CT (computed Tomography); CTGF (Connective Tissue Growth Factor); DNAH1 (Dynein Axonemal Heavy Chain 1); DNAH9 (Dynein Axonemal Heavy Chain 9); EMT (Epithelial–Mesenchymal Transition); FEV1 (Forced Expiratory Volume in the first second); FVC (Forced Vital Capacity); HRCT (High-Resolution Computed Tomography); ICS (Inhaled Corticosteroids); IFN-y (Interferon-gamma); IgG4 (immunoglobulin G4); IL (interleukin); LDH (Lactate Dehydrogenase); LL-37 (Cathelicidin antimicrobial peptide); MBL (Mannose-Binding Lectin); MMP (Matrix Metalloproteinase); MPO (Myeloperozidase); MUC5/MUC5B (Mucin 5/Mucin 5B); NOD2 (Nucleotide-binding Oligomerization Domain-containing protein 2); PDGF (Platelet-Derived Growth Factor); PIBO (Post-Infectious Bronchiolitis Obliterans); S100A4 (S100 calcium-binding protein A4); SARS-CoV-2 (Severe Acute Respiratory Syndrome Coronavirus 2); TGF-/TGF-1 (Transforming Growth Factor-beta/1); TNF-α (Tumor Necrosis Factor-alpha); Tregs (Regulatory T-cells); TSLP (Thymic Stromal Lymphopoietin); YKL-40 (chitinase-3-like protein 1).
